# The mediating role of proactive coping in the relationships between stress mindset, challenge appraisal tendencies, and psychological wellbeing

**DOI:** 10.3389/fpsyg.2023.1140790

**Published:** 2023-10-24

**Authors:** Paul C. Mansell, Martin J. Turner

**Affiliations:** ^1^School of Health, Science and Wellbeing, Staffordshire University, Stoke-on-Trent, United Kingdom; ^2^Manchester Metropolitan University, Manchester, United Kingdom

**Keywords:** proactive coping, REBT, stress beliefs, athlete mental health, stress appraisal

## Abstract

**Objective:**

Stress is ubiquitous and how individuals view the nature of stress can influence psychological wellbeing. The present study aimed to investigate the mediating role of proactive coping on the relationships between stress mindset and challenge appraisal tendencies and examine how this in turn related to psychological wellbeing. A secondary aim was to investigate if there were any differences in stress mindset between athletes and non-athletes. It was hypothesised that stress mindset would be indirectly positively associated with challenge appraisal tendencies through proactive coping, that a challenge appraisal tendency would positively relate to vitality, and that vitality would negatively relate to depressive symptoms. It was also hypothesised that athletes would possess more facilitative views of stress compared with non-athletes.

**Methods:**

Two hundred and seven individuals (*n* = 101 athletes, *n* = 106 non-athletes, *M*_age_ = 22.76 years, *SD* = 4.94) completed an online questionnaire pack assessing stress mindset, proactive coping, challenge appraisal tendencies, vitality, and depressive symptoms.

**Results:**

Using path analysis, the hypothesised model demonstrated a good fit to the data and the positive relationship between stress mindset and challenge appraisal tendencies was mediated by proactive coping. Challenge appraisal tendencies were positively associated with vitality, which was negatively associated with depressive symptoms. Athletes reported a significantly greater ‘stress-is-enhancing’ mindset, greater vitality, and fewer depressive symptoms than non-athletes.

**Conclusion:**

Findings offer support for the role that stress mindset has in potentially influencing psychological wellbeing and offer the novel suggestion that this mechanism may operate through proactive coping and challenge appraisal tendencies.

## Highlights

- Stress mindset was not related to challenge appraisal tendencies directly.
- Stress mindset related to challenge appraisal tendencies through proactive coping.
- Challenge appraisal tendencies related to vitality, which in turn related to depressive symptoms.
- Athletes reported a greater ‘stress-is-enhancing’ mindset than non-athletes.

## Introduction

Psychological distress, including conditions such as depression, is one of the main causes of disease worldwide (Vos et al., 2015). In the United Kingdom, psychological distress is reported to be a greater disease burden than both cancer and heart conditions (Mental Health Foundation, 2015) with one in four adults said to experience poor mental health during their lifetime (NHS, 2022). Depressive symptoms are a major contributor to psychological distress, with 21% of adults experiencing some form of depression at one time (Office for National Statistics, 2021).

One factor which is proposed to increase the risk of psychological distress is stress. Stress is experienced when the perceived demands of the environment outweigh the ability to cope (Cohen et al., 2007). Stressors can be in the form of events, situations, or environmental conditions where a potential negative impact is perceived by an individual (Halbreich, 2021). When stress is chronic and excessive, this increases an individual’s allostatic load and contributes significantly to psychological distress (Mücke, 2018), such as increased depressive symptoms (Cohen et al., 2007). Indicators of psychological wellbeing such as vitality (i.e., feeling alive and full of energy; Fruchart and Rulence-Pâques, 2020) are associated with lower levels of depressive symptoms (Ryan and Frederick, 1997). In athletes specifically, vitality is seen as a contributor to eudemonic wellbeing, and alongside high performance, this is supports thriving in their sporting pursuits (Brown et al., 2021). However, stress also relates to lower levels of vitality (Rozanski and Kubzansky, 2005). Consequently, stress is typically considered to be a deleterious construct. In support, one study found that 85% of participants reported stress to have a negative impact on health and productivity (McGonigal, 2016). Rather than automatically equating stress with distress (Rudland et al., 2020), it is possible to view stress and its consequences positively (Dixon et al., 2017), which may result in downstream psychological benefits (Laferton et al., 2019). This is particularly important as it is not possible to avoid stress entirely.

Rather than trying to eliminate stress, a growing body of work has begun to consider how our beliefs about the nature of stress may influence indicators of wellbeing such as vitality and depressive symptoms (e.g., Jiang et al., 2019). How depressive symptoms and vitality are related to beliefs about stress may be explained by Rational Emotive Behaviour Therapy (REBT; Ellis and Dryden, 2007). This framework posits that individuals’ beliefs influences their psychological wellbeing (Turner et al., 2019). According to the REBT framework, irrational beliefs (i.e., fixed, illogical and extreme) lead to psychological distress, and contrastingly, rational beliefs promote psychological wellbeing. Possessing irrational beliefs about the nature of stress (e.g., considering stress to always be negative and something to avoid) may therefore lead to psychological distress, although there is little previous research that has investigated these relationships. Consequently, efforts to investigate the indirect association between beliefs about stress and psychological wellbeing (Kilby et al., 2020) may shed more light on the mechanisms of such relationships and subsequently help individuals in dealing with stress.

As part of their sporting pursuits, athletes are a group of people who experience a wide range of stressors. This may include organisational stressors (e.g., disrupted sleep patterns) and personal stressors (e.g., maintenance of relationships; Rice et al., 2016), but it is competitive stressors that may particularly increase their experience of stress. In training and in competitive fixtures, athletes strive to meet the demands and expectations placed on themselves as well as others. They face de-selection, heightened risk of injury, and the risk of losing income that may be tied to their success, and stressors such as these may be exacerbated for those performing at elite levels (Fletcher et al., 2012). Athletes’ interpretations of stressful encounters are important in determining how they respond (Perry, 2020) and they may employ a range of self-regulation and coping strategies to enable them to achieve adaptive outcomes (Nicholls and Perry, 2016). This means that exploring how athletes’ beliefs about stress may contribute to their psychological wellbeing warrants attention for researchers (Didymus and Jones, 2021).

Our meta-beliefs about the nature of stress can be conceptualised as stress mindset (Jamieson et al., 2018). Stress mindset refers to the extent to which an individual believes that stress has enhancing or debilitating consequences (Crum et al., 2013). Those who perceive that stress can have positive consequences on stress-related outcomes, such as health, productivity and performance are said to possess a ‘stress-is-enhancing’ mindset, whilst those who view stress as a maladaptive construct possess a ‘stress-is-debilitating’ mindset (Crum et al., 2013). Rather than being two dichotomous states, individuals’ beliefs will sit somewhere along the stress mindset continuum, although it is thought that most individuals perceive stress to be debilitative (Crum et al., 2013). It is possible to alter stress mindset (e.g., Keech et al., 2021), and adopting facilitative views about stress may increase the likelihood of coping with demanding situations (Kim et al., 2020; Smith et al., 2020), perhaps due to the adaptive influence of stress mindset on stress appraisals (Kilby and Sherman, 2016). Holding a ‘stress-is-enhancing’ mindset is also reported to facilitate responses to stress (Park and Hahm, 2019), improve work productivity (Crum et al., 2013) and enhance academic performance (Keech et al., 2018). However, research in the domain of stress mindset is still fairly novel, and explorations continue as to how exactly it influences stress-related outcomes.

Beyond stress mindset, stress appraisal in the form of challenge and threat appraisals has also been found to relate to psychological wellbeing. Individuals may be predisposed to appraise ongoing relationships with the environment as either a challenge or a threat on a consistent basis (Lazarus, 1991). Those who believe that they possess the resources to cope with the demands of situations will likely experience challenge appraisal tendencies whilst the opposite is true of threat appraisal tendencies (Lazarus et al., 1980). Challenge appraisal tendencies are associated with strong coping expectancies and positive emotions, and in contrast, those who tend to adopt threat appraisal styles may be more likely to experience weak coping expectancies and negative emotions (Skinner and Brewer, 2002). Importantly, challenge appraisal tendencies may inform state challenge appraisals when confronted with stressors meaning that the more an individual exhibits a trait challenge appraisal tendency, the more likely they will be to appraise specific stressful situations as a challenge rather than a threat (Skinner and Brewer, 2002; Cumming et al., 2017). Additionally, challenge appraisals are related to lower levels of depression (Mak et al., 2004), positive affect (Chadha et al., 2019), and individuals who adopt challenge appraisals are more likely to mobilise increased energy for action (Carenzo et al., 2020), consistent with vitality (Lavrusheva, 2020). Hence, challenge appraisal tendencies are considered to have positive downstream influences on psychological wellbeing.

Despite their similarities in being associated with cognitions of stressful situations, distinctions exist between the concepts of stress mindset and challenge and threat appraisals (Crum et al., 2013). Stress mindset theory focuses on metacognitive beliefs about the nature of stress in general, and disregards contextual information about specific stressors (Crum et al., 2017). In contrast, appraisals are concerned with cognitive evaluations of stressors, which may be in relation to general appraisal styles (e.g., Cumming et al., 2017) or those of specific events (Kilby and Sherman, 2016). Therefore, adopting a ‘stress-is-enhancing’ mindset is not a guarantee of enhancing challenge appraisal tendencies, but adopting this mindset may contribute to cognitive, emotional, and behavioural responses that are adaptive when faced with stressful situations (Crum et al., 2017). However, due to their similarities, beliefs about the nature of stress (e.g., stress mindset) are thought to relate to the appraisals of specific stressful situations as a challenge or a threat (Jamieson et al., 2018).

The potential for stress mindset to relate to stress appraisals tendencies has been noted by Kilby and Sherman (2016). Furthermore, stress appraisals are thought to mediate the relationship between stress mindset and psychological wellbeing, however, there has been little research which has explored these associations at trait level. Mansell (2021) tested the associations between stress mindset, challenge appraisal tendencies, vitality, and depressive symptoms. Path analysis demonstrated that stress mindset was indirectly associated with vitality (positively) and with depressive symptoms (negatively), through challenge and threat appraisal tendencies. Specifically, a more ‘stress-is-enhancing’ mindset was associated with greater challenge appraisal tendencies which were associated with greater vitality. In turn, vitality was associated with lower depressive symptoms. Previous findings have suggested that stress mindset and threat appraisals may not be related during the absence of an imminent stressor (Kilby and Sherman, 2016). This may be due to the notion that challenge and threat appraisals are not necessarily two extremes at opposite ends of a scale, but two separate constructs (Evans et al., 2018), which suggests that it makes conceptual sense to assess the positive constructs separately to the negative constructs (Skinner and Brewer, 2002). As the study by Mansell (2021) was conducted with athletes, it is important to investigate whether similar findings are also replicated in non-athlete samples, and to how ascertain how the associations between stress mindset and challenge appraisal tendencies may occur.

Although some studies have reported a direct relationship between stress mindset and challenge appraisal tendencies (e.g., Mansell, 2021), other studies suggest that relationships between stress mindset and positive stress-related outcomes may be indirect (Kilby et al., 2020; Klussman et al., 2020) indicating inconsistencies within the literature. As those who possess adaptive mindsets often engage in facilitative coping strategies tend to experience positive outcomes (e.g., Yeager et al., 2016), it may be that the association between a ‘stress-is-enhancing’ mindset and challenge appraisal tendency could be due proactive coping. Considered a suitable method of preparing for confrontation with inevitable stressors (Serrano et al., 2021), proactive coping is characterised by perceiving risks and demands to be opportunities for growth and by taking constructive actions to deal with stressors (Greenglass and Fiksenbaum, 2009). This distinguishes proactive coping from other adaptive coping strategies as it refers to the accumulation of resources and strategies before a stressor is present (Greenglass and Fiksenbaum, 2009). The accumulation of these personal resources provides individuals with greater feelings of control and optimism (Aspinwall and Taylor, 1997), leading to approach-type behaviours (e.g., problem-solving; Devonport et al., 2013) indicative of challenge appraisals (Jones et al., 2009). Indeed, employees who reported a ‘stress-is-enhancing’ mindset were found to use more approach-coping efforts when faced with a high workload, which was also associated with greater vigour and task performance (Casper et al., 2017). Accordingly, this may explain the known association between proactive coping and challenge appraisals (Raper and Brough, 2021) as individuals experience a greater sense of control. In addition to associations with stress mindset (e.g., Keech et al., 2018), proactive coping has also been found to be negatively associated with depressive symptoms (Wagner and Martin, 2012). This adds to the suggestion that proactive coping may play an important role in influencing stress-related outcomes. However, despite coping’s inseparability from stress appraisals (Tamminen, 2021), little is known about how proactive coping specifically may contribute to challenge appraisal tendencies.

Based on recent studies (e.g., Keech et al., 2018), it is likely that stress mindset predicts proactive coping. Both stress mindset and proactive coping consist of general beliefs before stress has occurred (Greenglass and Fiksenbaum, 2009) rather than beliefs about a particular situation (Wagner and Martin, 2012), and they comprise of realistic and flexible thought processes (Lazarus and Folkman, 1984). This relationship between the two may be explained using the ABC framework of REBT (Ellis and Dryden, 2007) in that individuals who possess a ‘stress-is-enhancing’ mindset may believe that it is not an adverse situation (A) that leads to behavioural and emotional consequences (C), but rather their beliefs about the situation (B). This ABC approach to cognitions (likely displayed by someone with a greater ‘stress-is-enhancing’ mindset) is said to enhance proactive coping (Wood and Turner, 2020). Furthermore, individuals who possess a ‘stress-is-enhancing’ mindset may view stress as a challenge (Guo et al., 2017), which is symptomatic of individuals who adopt proactive coping strategies (Pirkkalainen et al., 2019). Taken together, it can be suggested that the relationship between stress mindset and challenge appraisal tendencies may operate through proactive coping.

Previous experiences play an important role in how individuals view stress (Holmes and Rahe, 1967). For instance, athletes may have accumulated experiences of stressful situations, such as regular competition (Perry, 2020). Although exposure to more severe stressful experiences can be detrimental to athletes (McLoughlin et al., 2021), athletes may develop tendencies to view stress (e.g., competitive situations) in a more positive light when compared to non-athletes through regular exposure to moderately stressful situations (Fletcher and Arnold, 2021) and subsequently, a more facilitative view of stress may be developed. Furthermore, through regular exposure to stress, athletes can engender ‘stress inoculation’ and learn strategies to cope with stress and consequently perceive stressful situations to be an opportunity rather than a threat (Turner et al., 2013). In support of this notion, Mansell (2021) found mean stress mindset scores of athletes to appear higher than those of non-athletes reported in other studies (e.g., Crum et al., 2013; Kilby and Sherman, 2016). This suggests athletes may have a more ‘stress-is-enhancing’ mindset than non-athletes. However, the paucity of stress mindset research in athletes means this this yet to be sufficiently examined and research is yet to directly compare stress mindset in athletes to that in non-athletes.

### Aims and hypotheses

**Figure 1:**
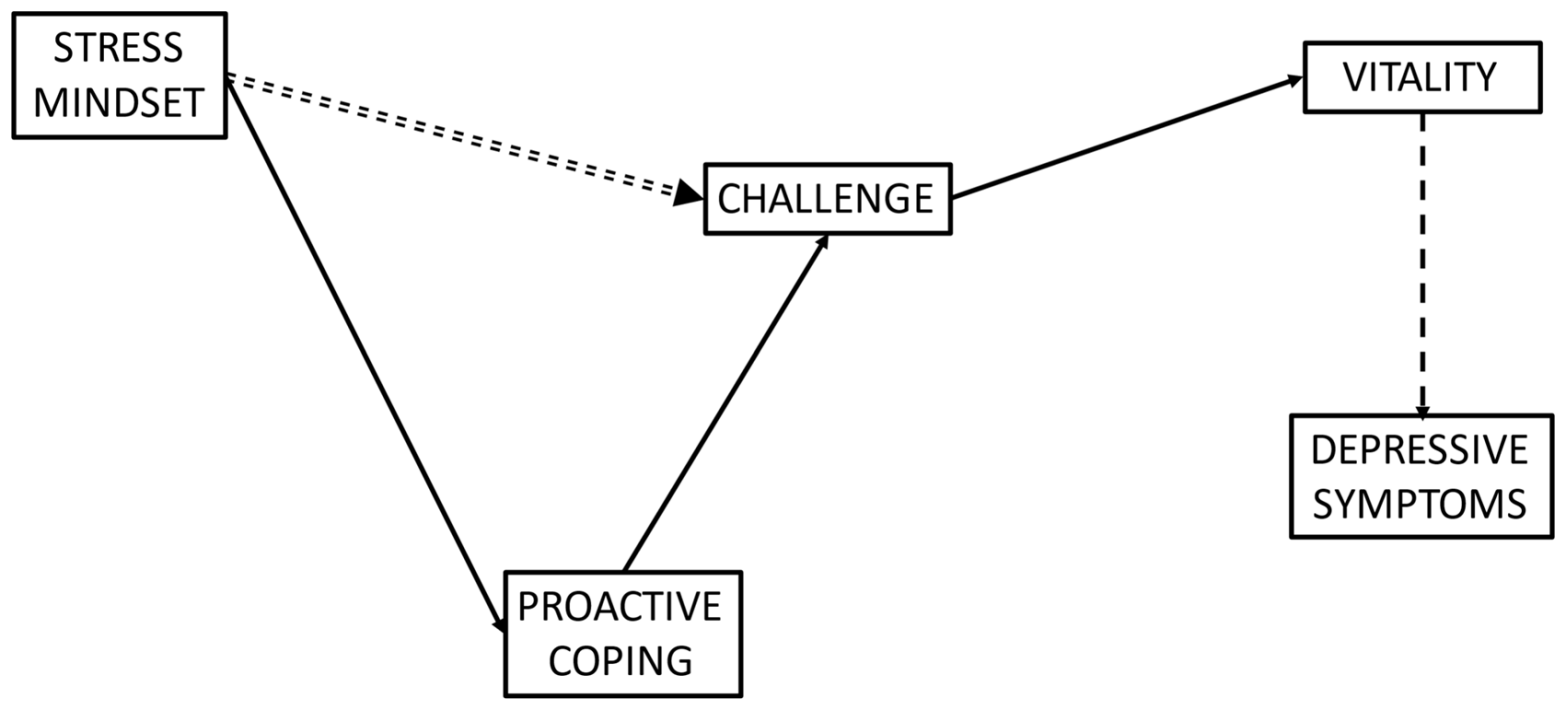
Hypothesised model. Dashed lines represent negative associations and unbroken lines represent positive associations. Double-dashed lines represent a non-significant path. For visual simplicity, control variables are not displayed.

Despite the apparent relationships between stress mindset and challenge appraisal tendencies (e.g., Mansell, 2021), and challenge appraisal tendencies with both vitality and depressive symptoms, research has yet to understand how stress mindset relates to challenge appraisal tendencies, and the subsequent relationships with vitality and depressive symptoms. Based on the identified associations between stress mindset and proactive coping (Keech et al., 2018), and proactive coping with challenge appraisal (Raper and Brough, 2021), it seems logical to suggest that proactive coping may mediate the relationship between stress mindset and challenge appraisal tendencies. Furthermore, it was hypothesised that challenge would be positively related to vitality, which in turn would negatively relate to depressive symptoms. Therefore, the aims of the present study were to investigate the extent to which proactive coping mediated the relationship between stress mindset and challenge appraisal tendency, and examine how this in turn related to vitality and depressive symptoms. The hypothesised model was based on that demonstrated by Mansell (2021) in which stress mindset was positively related to challenge appraisal tendencies, and challenge appraisal tendencies were positively related to vitality, whilst vitality was negatively related to depressive symptoms. However, proactive coping was also proposed to explain the relationship between stress mindset and challenge appraisal tendencies by fully mediating the relationship between the two variables. Therefore, it was hypothesised that stress mindset would be positively associated with proactive coping which in turn would be positively associated with a greater challenge appraisal tendency. Consequently, it was also hypothesised that the direct association between stress mindset and challenge appraisal tendency would be non-significant and instead an indirect positive association between stress mindset and challenge appraisal tendency would operate through proactive coping. In line with findings by Mansell (2021), it was predicted that challenge appraisal tendency would positively relate to vitality, and vitality would negatively relate to depressive symptoms. The hypothesised model is displayed in Figure 1. A secondary aim of the present study was to investigate whether there were any differences in stress mindset between athletes and non-athletes. It was hypothesised that athletes would hold a greater ‘stress-is-enhancing’ mindset compared with non-athletes.

## Methods

### Participants

Two hundred and seven individuals (*n* = 153 females, *n* = 53 males, *n* = 1 genderfluid, *M*age = 22.76 years, *SD* = 4.94) participated in the study. The sample consisted of a mixture of athletes (*n* = 101) and non-athletes (*n* = 106). Athletes stated that they regularly took part in sport ranging in competitive level from recreational (*n* = 32), through to club (*n* = 47), and regional and above (*n* = 27). Exclusion criteria included not having any medically diagnosed mental health conditions at the time of taking part in the study, whilst inclusion criteria were that individuals were aged 18–35, proficient in reading English, and had access to the internet. Ethical approval was granted from the author’s university ethics committee before advertisements were launched to recruit participants on campus and throughout the United Kingdom via social media channels such as Facebook and Twitter.

### Measures

#### Stress mindset

Stress mindset was assessed using the 8-item unidimensional Stress Mindset Measure—General (SMM-G; Crum et al., 2013). Four statements emphasise more of a stress-is-enhancing mindset (e.g., *‘Experiencing stress enhances my learning and growth’*), and four statements represent a more stress-is-debilitative mindset (e.g., *‘Experiencing stress depletes my health and vitality’*). Participants rated the extent that they agreed with each of the eight statements on a 5-point Likert scale ranging from 0 (*strongly disagree*) to 4 (*strongly agree*). The four negatively worded items are reverse scored before all 8 items are averaged together on one subscale. A higher value reflects that an individual possesses a more ‘stress-is-enhancing’ mindset. The SMM-G has been used in other similar studies (e.g., Mansell, 2021) and has been reported to produce valid and reliable stress mindset scores (Crum et al., 2017). The present study demonstrated good internal reliability using Cronbach alpha’s coefficient (0.82).

#### Proactive coping

The Proactive Coping Scale (PCS; Greenglass et al., 1999) was employed to assess the extent to which an individual’s cognitions and behaviours are reflective of proactive coping (Greenglass et al., 1999). The PCS is a subscale from the multidimensional Proactive Coping Inventory (Greenglass et al., 1999) and has been reported to be a valid and reliable measure (Sohl and Moyer, 2009), with use in other similar studies involving psychological wellbeing (e.g., Wagner and Martin, 2012). Fourteen items (e.g., *‘I turn obstacles in to positive experiences’*) form the PCS and participants select the extent to which they agree with each statement on a 4-point Likert scale ranging from 1 *(not at all true)* to 4 *(completely true)*. Negatively worded items are reverse scored before all items are summed, with higher scores indicating a greater tendency to proactively cope. The Cronbach alpha coefficient in the present study was 0.85, indicating good levels of internal reliability.

#### Challenge appraisal tendency

The challenge subscale of the Cognitive Appraisal Scale (CAS; Skinner and Brewer, 2002) was used to assess the extent to which individuals tend to appraise meaningful situations as a challenge (e.g.*, ‘Overall I expect that I will achieve success rather than experience failure’*). The CAS is an 18-item questionnaire with 8 items assessing challenge appraisal tendency and 10 items assessing threat appraisal tendency. Participants indicate the extent to which they agree or disagree with each statement by responding on a 6-point Likert scale ranging from 1 (*strongly disagree*) to 6 (*strongly agree*). For the purpose of the present study only the challenge subscale was used. Mean scores were generated so a higher score indicated a greater challenge appraisal tendency. The Cronbach alpha coefficient in the present study was 0.75, indicating acceptable levels of internal reliability. The CAS provides valid and reliable challenge appraisal tendencies scores and has been used in other similar studies (Williams and Cumming, 2012).

#### Vitality

Participants’ feelings of positive affect and personal energy were assessed using the Subjective Vitality Scale (SVS; Ryan and Frederick, 1997). Seven items are included in total (e.g., *‘I have energy and spirit’*) in which participants indicate the extent to which each statement reflects their views about their life in general. Responses are made on a 7-point Likert scale ranging from 1 (*not at all true*) to 7 (*very true*). One item (‘*I do not feel very energetic*’) was reverse scored, and all items are then summed so that a higher score indicates a greater subjective vitality. The Cronbach alpha coefficient in the present study was 0.91, indicating high levels of internal reliability. The SVS has been found to be a valid and reliable measure of vitality (Mansell, 2021) and has been used other recent studies that have investigated psychological wellbeing (Davis and Turner, 2020).

#### Depressive symptoms

The depressive symptoms subscale of the Hospital Anxiety and Depression Scale (HADS; Zigmond and Snaith, 1983) was used to assess depressive symptoms. This subscale is made up of 7 out of 14 HADS items. For each item (e.g., ‘*I feel cheerful*’ and ‘*I look forward with enjoyment to things*’) participants are asked to consider which reply comes closest to describing how they have been feeling over the last 2 weeks. Responses are made on a 4-point Likert scale ranging from 0 to 3 and anchors are worded slightly differently depending on the item. For example, in response to ‘*I feel as if I am slowed down*,’ participants can select responses ranging from 0 = ‘*Not at all’ to* 3 = *‘Nearly all the time.’* Positively worded items are reverse scored, and scores are then summed with higher scores indicating higher levels of depressive symptoms. The HADS has been found to be a valid and reputable measure of depressive symptom severity (Zigmond and Snaith, 1983) and has been used recently in other studies (e.g., Weber et al., 2018). The Cronbach alpha coefficient in the present study was 0.77, indicating acceptable levels of internal reliability.

### Procedures

Data collection took place for 5 months from October 2020 to February 2021. Potential participants were provided with an information sheet about the study, including inclusion/exclusion criteria, and details of key ethical considerations such as data confidentiality and their freedom to withdraw at any time. Those individuals agreeing to take part provided informed consent before completing an online questionnaire pack obtaining some demographic and sport information (if they played a sport) and containing the SMM-G, PCS, CAS, SVS, and HADS. The questionnaire pack took around 20 min to complete, and participants were thanked for taking part in the study upon completion.

### Data availability and analyses

The data that support the findings of this study are available from the corresponding author upon reasonable request. Data were screened and cleaned in SPSS (IBM, version 27). The data were found to have less than 5% of missing responses. Little’s MCAR Test was employed to confirm that this data was missing completely at random (*p* > 0.05), and accordingly, the expectation maximisation method was utilised as a suitable method to complete the missing data (Tabachnick and Fidell, 2013). Next, data were checked for outliers and normality. Checks with boxplots revealed no significant univariate outliers, and no multivariate outliers were discovered when using Mahalanobis distance at *p* < 0.001 (Tabachnick and Fidell, 2013), so all data were retained for the analysis. All normality tests met the assumptions necessary for parametric data analysis.

To check that the questionnaire data was valid and reliable, confirmatory factor analysis (CFA) using AMOS (version 27) was conducted on all questionnaires to assess fit indices for all the questionnaires (see Table 1). Cronbach alpha co-efficients were conducted on all questionnaire subscales along with chi-square (χ^2^; Jöreskog and Sörbom, 1993) and degrees of freedom, comparative fit index (CFI), Goodness of Fit index (GFI), root mean square error of approximation (RMSEA), and standardised root mean square residual (SRMR). In cases of poor model fit, suggestions of modification indices were followed (Tabachnick and Fidell, 2013). Accordingly, CFA results suggest that the measures used in the present study are valid and reliable (e.g., Hair et al., 2010).

**Table 1:** Confirmatory factor analysis fit indices for the questionnaires employed the in the study.

	*x^2^ (df)*	CFI	GFI	SRMR	RMSEA (90% CI)
SMM-G	29.57 (13)	0.966	0.966	0.043	0.079 (0.041–0.117)
PCS	95.69 (68)	0.968	0.935	0.047	0.045 (0.020–0.064)
CAS (challenge)	27.65 (17)	0.966	0.969	0.041	0.055 (0.003–0.091)
HADS (depressive symptoms)	19.02 (77)	0.986	0.973	0.039	0.042 (0.000–0.085)
SVS	38.96 (12)	0.973	0.952	0.031	0.105 (0.069–0.142)

One-way ANOVAs were conducted to see whether there were any gender and athlete vs. non-athlete differences in the different variables of interest (i.e., stress mindset, proactive coping, challenge appraisal tendency, vitality, and depressive symptoms) due to differences emerging in previous research (e.g., Mak et al., 2004; Ouwehand et al., 2008). Participants who identified their gender as ‘genderfluid’ were not included in this analysis due to the number of people in this group not being comparable to the numbers in the other groups to conduct the relevant analyses of variance. Pearson’s correlations were also conducted to test for associations between age and the variables of interest. These findings were used to identify whether gender, sport participation, and age should be controlled for when testing the hypothesised model.

The hypothesised model was tested using path analysis in AMOS (version 27). Kline’s (1998) recommendations for the number of participants to complete path analysis were adhered to with 207 participants considered as adequate to proceed. To determine whether stress mindset was associated with challenge appraisal via proactive coping, pathways were inserted from stress mindset to proactive coping and from proactive coping to challenge appraisal. The direct pathway between stress mindset and challenge appraisal was also added with the hypotheses being that this would be non-significant due to the relationship operating via proactive coping. Based on previous research, pathways were also inserted from challenge appraisal to vitality, and from vitality to depressive symptoms. Based on previous findings (e.g., Mak et al., 2004), gender and sport participation were controlled for in the analysis. The hypothesised model is displayed in Figure 1. The model’s goodness of fit was examined using the chi square likelihood statistic ratio (χ^2^; Jöreskog and Sörbom, 1993). Additionally, the CFI and the TLI were used as measures of incremental fit, with values of ≥0.95 and ≥ 0.90 demonstrating an excellent model fit (Hu and Bentler, 1999). Furthermore, the RMSEA and SRMR were chosen as indices of absolute model fit, where criteria of ≤0.05 and ≤0.08 reflected excellent and adequate model fit, respectively (Hu and Bentler, 1999; Byrne, 2010). Similar measures of model fit were also used in other comparable studies (e.g., Chadha et al., 2019). In cases of poor model fit, modification indices were examined and meaningful covariances with larger regression weights were considered and included into subsequent iterations of the proposed model (Byrne, 2010). Standardised regressions were reported for all direct and indirect effects. Indirect effects were examined using 95% bias-corrected confidence intervals generated from bootstrapping of 1,000 samples.

## Results

### Descriptive statistics, gender differences, and sport participation differences

Participant means and standard deviations of stress mindset, proactive coping, challenge appraisal tendency, vitality, and depressive symptoms for the sample as a whole and broken down for males and females and sport participation are displayed in Table 2. One-way ANOVA results revealed that males recorded significantly higher proactive coping, *F*(1, 205) = 6.44, *p* = 0.012, η_p_^2^ = 0.03, and challenge appraisal, *F*(1, 205) = 6.73, *p* = 0.001, η_p_^2^ = 0.06, compared with females. There were no significant gender differences in stress mindset, vitality, or depressive symptoms. Further one-way ANOVA results revealed that athletes reported a significantly greater ‘stress-is-enhancing’ mindset *F*(1, 205) = 8.93, *p* = 0.003, η_p_^2^ = 0.04 and higher vitality *F*(1, 205) = 7.95, *p* = 0.005, η_p_^2^ = 0.04 compared with non-athletes, whilst non-athletes recorded significantly higher depressive symptoms *F*(1, 205) = 8.49, *p* = 0.004, η_p_^2^ = 0.034 compared with athletes. There were no significant sport participation differences in proactive coping or challenge appraisal tendencies.

**Table 2:** Participant characteristics, male and female differences, and sport participation differences in stress mindset, proactive coping, challenge, depressive symptoms and vitality.

	Overall sample mean (SD)	Males mean (SD)	Females mean (SD)	Athletes mean (SD)	Non-athletes mean (SD)
Stress mindset	1.88 (0.63) CI (1.80–1.97)	2.03 (0.70) CI (1.83–2.22)	1.83 (0.60) CI (1.74–1.93)	2.01^**^ (0.60) CI (1.89–2.12)	1.75 (0.64) CI (1.62–1.88)
Proactive coping	2.92 (0.44) CI (2.86–2.98)	3.05^**^ (0.44) CI (2.93–3.17)	2.88 (0.44) CI (2.81–2.95)	2.96 (0.60) CI (2.88–3.04)	1.75 (0.60) CI (2.79–2.98)
Challenge	4.41 (0.60) CI (4.33–4.49)	4.65^**^ (0.58) CI (4.49–4.81)	4.33 (0.59) CI (4.23–4.42)	4.45 (0.62) CI (4.33–4.58)	4.36 (0.55) CI (4.25–4.47)
Vitality	28.69 (8.64) CI (27.50–29.88)	30.06 (8.84) CI (27.62–32.49)	28.21 (8.55) CI (26.85–29.58)	30.31^**^ (8.14) CI (28.74–31.88)	26.97 (8.85) CI (25.21–28.73)
Depressive symptoms	4.87 (3.53) CI (4.39–5.36)	4.28 (3.15) CI (3.41–5.15)	5.08 (3.65) CI (4.50–5.66)	4.19^**^ (2.85) CI (3.64–4.74)	5.60 (4.03) CI (4.80–6.40)

^**^p < 0.005 level (sig. 2). Participants who identified their gender as ‘genderfluid’ were not included in this analysis due to the number of people in this group not being comparable to the numbers in the other groups to conduct the relevant analyses of variance. Confidence Interval (CI) 95%.

### Associations with age

Correlation analysis of the extent to which age was associated with stress mindset, proactive coping, challenge appraisal tendency, vitality, and depressive symptoms are displayed in Table 3. The present study found no significant correlations between age and any of the variables of interest (*p*’s ≥ 0.12). As such, only gender and sport participation were controlled for in the hypothesised model.

**Table 3:** Age correlations with stress mindset, proactive coping, challenge, depressive symptoms and vitality.

	Pearson’s correlation (r)
Stress mindset	−0.054
Proactive coping	−0.051
Challenge	−0.109
Depressive symptoms	0.060
Vitality	−0.096

### Model

**Figure 2:**
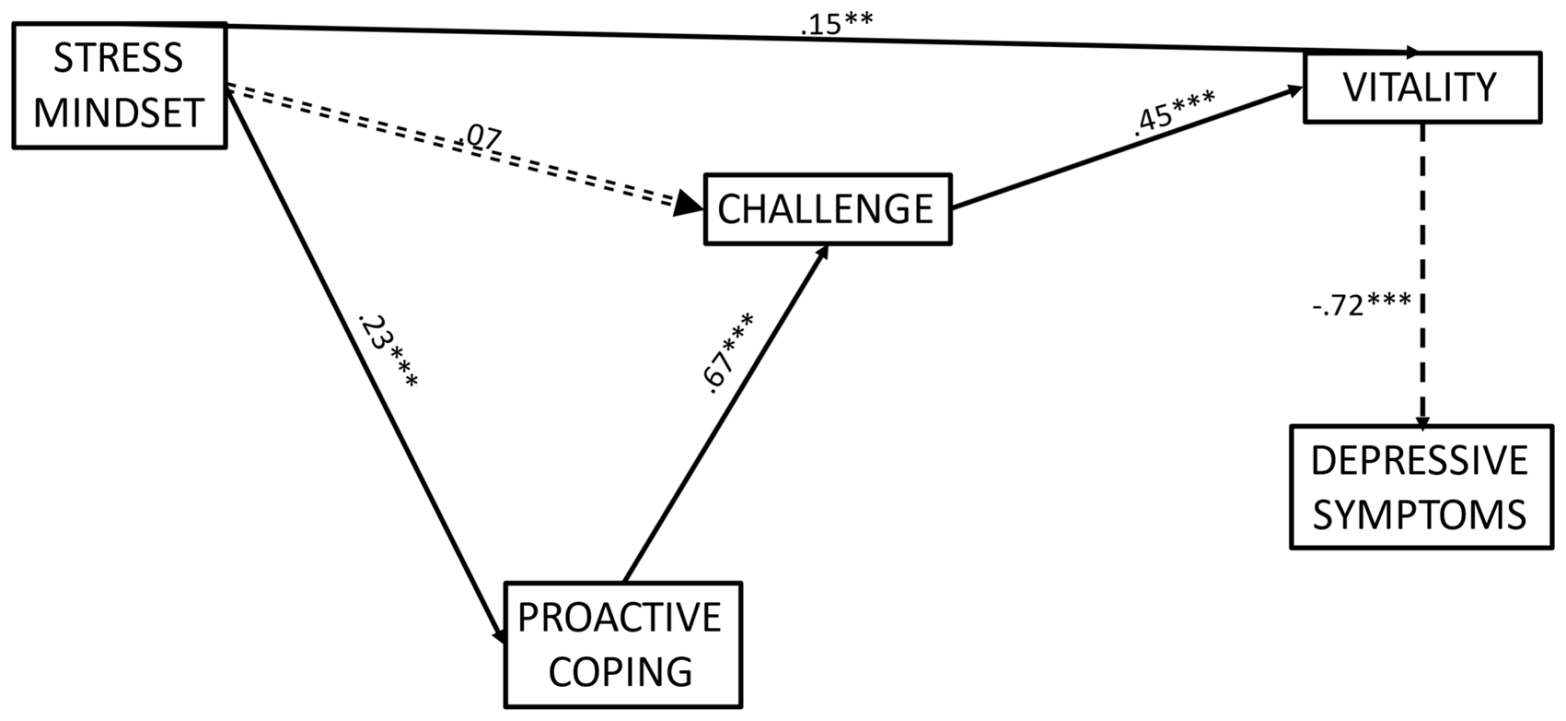
Final model displaying the effect of stress mindset on challenge appraisal tendency mediated by proactive coping, and on depressive symptoms and vitality through proactive coping and challenge appraisal tendency. Numbers refer to standardised beta values. ^*^p < 0.05, ^**^p < 0.01, ^***^p < 0.001. For visual simplicity, control variables are not displayed.

Path analysis revealed that the hypothesised model demonstrated a good fit to the data *χ*^2^(7) = 28.30, *p* < 0.05, GFI = 0.96, TLI = 0.84, RMSEA = 0.12 (CI = 0.08 to 0.17) SRMR = 0.07. However, modification indices recommended an additional direct pathway from stress mindset to vitality. This pathway was considered to make sense conceptually and was subsequently added to the hypothesised model. Indeed, given that vitality is associated with subjective feelings of psychological and physiological energy (Lavrusheva, 2020), it is plausible to suggest that possessing a ‘stress-is-enhancing’ mindset may directly lead to higher vitality as well as through proactive coping and challenge appraisal tendency. Following the addition of this pathway, the revised model demonstrated an improved and good fit to the data *χ*^2^(6) = 22.72, *p* = 0.001, GFI = 0.97, TLI = 0.85, RMSEA = 0.12 (CI = 0.07 to 0.17), SRMR = 0.06. The standardised path coefficients for each individual path are displayed in Figure 2. Stress mindset was positively associated with proactive coping (*p* < 0.001), accounting for 5% of the variance. In turn, proactive coping was positively associated with challenge appraisal tendency (*p* < 0.001) accounting for 45% variance, thus mediating the non-significant relationship between stress mindset and challenge appraisal tendency. A non-significant direct path was found between stress mindset and challenge appraisal tendency. Additionally, challenge appraisal tendency was positively associated with vitality (*p* < 0.001), accounting for 20% variance, whilst vitality was negatively associated with depressive symptoms (*p* < 0.001), accounting for 52% variance.

Results of the indirect effects demonstrated that stress mindset had a significant indirect relationship with challenge appraisal tendency (β = 0.15, *p* = 0.023, 95% CI = 0.06 to 0.26) through proactive coping. Stress mindset also had a significant indirect relationship with vitality (β = 0.10, *p* = 0.005, 95% CI = 0.05 to 0.27) through proactive coping and challenge appraisal tendency. Furthermore, stress mindset also indirectly related to depressive symptoms (β = −0.18, *p* = 0.023, 95% CI = −0.28 to −0.06) through proactive coping, challenge appraisal tendency, and vitality. Proactive coping was found to have a significant indirect effect on vitality (β = 0.30, *p* = 0.023, 95% CI = 0.19 to 0.41) through challenge appraisal tendency, and on depressive symptoms (β = −0.22, *p* = 0.015, 95% CI = −0.29 to −0.13) through challenge appraisal tendency and vitality. Finally, challenge appraisal tendency was reported to have a significant indirect effect on depressive symptoms (β = −0.32, *p* = 0.030, 95% CI = −0.40 to −0.20) through vitality.

## Discussion

The aims of the present study were to investigate the extent to which proactive coping mediated the relationship between stress mindset and challenge appraisal tendency and examine how this in turn related to vitality and depressive symptoms. In support of the hypothesis, stress mindset was positively associated with proactive coping, which was in turn positively associated with a greater challenge appraisal tendency. Moreover, the direct association between stress mindset and challenge appraisal tendency was non-significant, supporting the hypothesised indirect positive association between stress mindset and challenge appraisal tendency through proactive coping. Although previous studies have found that stress mindset is positively associated with challenge appraisal tendency (e.g., Mansell, 2021), the findings of the present study extend the literature by demonstrating an apparent mechanism through which this relationship operates.

The positive association between stress mindset and proactive coping supports the work by Keech et al. (2018) and may be explained through the REBT framework (Ellis and Dryden, 2007). To elaborate, REBT proposes that it is not an adverse situation (A) in itself that leads to behavioural and emotional consequences (C), but rather an individual’s beliefs about the situation (B). Often, an individual’s behavioural and emotional consequences (C) are a direct result of an adverse situation (A) (i.e., A ➔ C). However, an individual’s beliefs about an adverse situation can influence the way in which they respond (i.e., A ➔ B ➔ C) which can lead to more positive approaches to stressful situations. Thus, individuals who hold more of the belief that ‘stress-is-enhancing’ can apply such beliefs (B) to adverse situations (A), leading to more flexible and adaptive responses including coping tendencies (C). Indeed, this A ➔ B ➔ C approach to thinking is said to lead to higher proactive coping (Wood and Turner, 2020). To illustrate, individuals who agree with items from the SMM-G (Crum et al., 2013) such as *‘Experiencing stress facilitates my learning and growth’* and possess a ‘stress-is-enhancing’ mindset (B) are more likely to respond in a proactive way (C) to cope with adverse situations (A).

The flexible and adaptive responses of proactive coping may explain the positive association it has with challenge appraisal tendencies. This finding is in accordance with research by Raper and Brough (2021), who demonstrated that proactive coping allows individuals to prepare for future events by developing their skills or accumulating personal resources. In turn, this likely increases their resource appraisals and may subsequently lead to a challenge appraisal tendency. For example, items on the PCS (Greenglass et al., 1999) such as *‘When I experience a problem, I take the initiative in resolving it’* appear to be closely aligned with the approach-focused behaviours suggested as indicative of challenge appraisals within the TCTSA (Jones et al., 2009). When considered from an REBT viewpoint, this may mean that challenge appraisal tendencies are a secondary behavioural and emotional consequence (C) of a ‘stress-is-enhancing’ mindset (B). Indeed, the actions and cognitions associated with proactive coping may enhance an individual’s perceived resources in respect of stressful situations, which is said to be an antecedent of challenge appraisals (Jones et al., 2009). Furthermore, the relationships between stress mindset, proactive coping, and challenge appraisal tendency supports theoretical concepts suggested within the TCSTA-R (Meijen et al., 2020) in that trait beliefs are important in determining challenge appraisals. That said, it may not always be the case that challenge appraisals will be experienced as a result of these beliefs and coping strategies because each individual and stressful situation are different. Overall, the findings suggest that a ‘stress-is-enhancing’ mindset may fuel proactive coping strategies that occur prior to stressful situations, and this may develop challenge appraisal tendencies. Subsequently, when stressful situations arise, an individual is more likely to appraise such events as a challenge and not as a threat.

It was predicted that challenge appraisal tendency would positively relate to vitality, and vitality would negatively relate to depressive symptoms. The results of the present study support these specific hypotheses and replicate the findings by Mansell (2021), whilst extending the literature by examining these relationships in a mixed sample of athletes and non-athletes. Associations, between challenge appraisals and psychological wellbeing have previously been established (e.g., Skinner and Brewer, 2004). The present study focuses on vitality as an indicator of psychological wellbeing, and perhaps an explanation for the relationship between challenge appraisal tendency and vitality is through feelings of higher energy. Previous studies have suggested this as a reason for this association (e.g., Carenzo et al., 2020), which supports cognitive appraisal theories of stress in that cognitive appraisals influence physiological responses (Tomaka et al., 1997). Psychologically, it may be that the generation of positive emotions resulting from challenge appraisals (Skinner and Brewer, 2004) means that an individual feels alert, energised and optimistic (Lavrusheva, 2020). Subsequently, these positive feelings associated with vitality may act as a buffer against the intensity of depressive symptoms that an individual experiences (Ryan and Frederick, 1997). The findings of the present study therefore suggest that positive stress appraisal tendencies are related to negative mental health indicators (i.e., depressive symptoms) through positive mental health indicators (i.e., vitality).

The secondary aim of the present study was to examine whether there were any differences between athletes and non-athletes in stress mindset. In support of the hypothesis, athletes reported a more ‘stress-is-enhancing’ mindset compared with non-athletes. This is the first known study to compare differences in stress mindset between athletes and non-athletes, although previous studies have reported seemingly higher mean scores in stress mindset in an athlete-only sample (Mansell, 2021) compared to a general population sample (e.g., Crum et al., 2013), thus suggesting a more ‘stress-is-enhancing’ mindset in athletes. The present study confirms this appears to be the case. A possible explanation for this distinction in athletes and non-athletes’ stress mindset is that athletes may have accumulated a greater number of opportunities to experience stressful situations. On a regular basis in both training and competitive situations, demands of high performance are placed on athletes (Perry, 2020). In addition to life’s daily demands, this exposes them to frequent situations of pressure—situations in which they will accumulate experiences they consider to be a success (e.g., winning, performing well). Therefore, a curvilinear relationship between stress and positive outcomes may be evident whereby frequent exposure to moderate levels of athletic-based stressful situations may lead to facilitative stress-related outcomes (Fletcher and Arnold, 2021). As such, these successful stressful experiences are likely to lead to developing beliefs that stress can be enhancing—particularly for performance and productivity—that are likely manifested in a more ‘stress-is-enhancing’ mindset compared to non-athletes. Perhaps the greater ‘stress-is-enhancing’ mindset partially explains the significantly better psychological wellbeing that was reported by athletes compared with non-athletes.

Furthermore, athletes’ more regular exposure to stressful situations compared with non-athletes may also act as a form of stress inoculation (Turner et al., 2013), whereby factors that may increase the perception of situations as stressful, such as novelty and uncertainty (Lazarus and Folkman, 1984) can be ameliorated. This explanation may be reflective of resilience, whereby regular exposure to stressful situations can lead to individuals perceiving stressful situations to be more manageable (Seery, 2011), thus aiding the development of a ‘stress-is-enhancing’ mindset. In the present study, the sample of athletes was drawn from a varied pool ranging from recreational, regional and above, and it may be that athletes pursuing their sport at higher levels experience a greater degree of pressure than recreational athletes (Fletcher et al., 2012). Accordingly, future research may wish to investigate whether there are significant differences in stress mindset in athletes at elite levels compared with recreational athletes. Research of this nature may help to explain if athletes develop a ‘stress-is-enhancing’ mindset *because* of their athletic pursuits.

Given the ubiquity of stress and the known potential for stress to have deleterious effects on psychological wellbeing, the findings of the present study may offer insight for practitioners who work in stress-related fields. Although the findings of the present study are cross-sectional and do not suggest causation, results imply that a ‘stress-is-enhancing’ mindset may trigger a set of cognitions and appraisals that could positively influence psychological wellbeing. This said, it is important to note that individuals should not seek to encounter a greater frequency and intensity of stress to experience the benefits associated with a ‘stress-is-enhancing’ mindset (Crum et al., 2013). Instead, the development of a ‘stress-is-enhancing’ mindset may be expedited by promoting rational beliefs about stress and reframing stressful experiences as useful learning opportunities rather than something to avoid (e.g., Jamieson et al., 2018). Providing opportunities for individuals to thrive in stressful situations (e.g., sporting competitions) and reflect on how their stress responses may have facilitated coping (Tamminen, 2021) may be fruitful in developing a ‘stress-is-enhancing’ mindset. Given that prolonged exposure to intense stressors may lead to psychological illbeing (Casper et al., 2017), practitioners who wish to enhance positive beliefs about stress should present that stress *can be* enhancing rather than is *wholly* enhancing (Keech et al., 2021). The novel aspect of the present study was the finding that the stress mindset is related to challenge appraisal tendencies indirectly through proactive coping. Accordingly, practitioners may wish to implement interventions that not only focus on the upsides of stress but also promote the use of proactive coping strategies prior to experiencing stress. This may include strategies that enhance perceptions of control by taking charge of stressful situations, or by encouraging the deployment of problem-solving strategies prior to stressful situations (Greenglass and Fiksenbaum, 2009). Results of the present study imply that this may lead to greater challenge appraisal tendencies and psychological wellbeing.

A strength of the present study was the relatively even samples of athletes (*n* = 106) and non-athletes (*n* = 101) enabling a comparison between the two groups in mindset. Furthermore, a cross-section of athletic ability was represented in the study’s sample. Future research may wish to consider investigating whether elite athletes possess differing levels of stress mindset to recreational athletes. As used in other similar studies (e.g., Çelik and Köse, 2021), the use of path analysis may be considered as a strength of the present study as it accounts for multiple associations simultaneously. Path analysis also examines direct and indirect pathways, which was important to test the indirect effect of stress mindset on challenge appraisal through proactive coping. The visual representation of the model also allows for clear representation of how the variables relate (Byrne, 2010). Future research may wish to use full Structural Equation Modelling with a larger sample size by using latent variables to explore a full model.

A limitation of the present study is that it is cross-sectional and does not imply causation. Consequently, it is important future research investigates whether interventions designed to enhance stress mindset subsequently result in increases in proactive coping and challenge appraisal tendencies, and whether these changes also result in enhanced performance in stressful situations as well as greater vitality and lower depressive symptoms. As the present study measured trait beliefs and appraisals and not those before an imminent stressor, future research could conduct similar measures immediately before a situation of pressure (Kilby et al., 2020). A study design of this nature may have more applied use as to how individuals prepare to face stressful situations. Future research may also wish to complement the use of psychological data with the addition of physiological or qualitative data immediately before a stressful event, such as a sporting competition. Indeed, as the data for this study was collected during the COVID-19 pandemic, it may be that this had an impact on results and influenced the responses of athletes specifically due to the additional stressors experienced during this period (Arnold and Fletcher, 2021). Additionally, future research may wish to combine measures of eudemonic wellbeing with hedonistic wellbeing (i.e., positive affect) to fully capture psychological wellbeing (e.g., Brown et al., 2017). The present study excluded individuals who had medically diagnosed mental health conditions, but future research may wish to investigate whether these results are replicated in individuals who have a clinical diagnosis of anxiety, for example. Moreover, future research may wish to assess whether the findings of the present study are also present in adults over the age of 35.

In conclusion, the present study aimed to investigate the extent to which proactive coping mediated the relationship between stress mindset and challenge appraisal tendency and examined how this in turn related to vitality and depressive symptoms. Using path analysis, data supported the model whereby holding more facilitative views of stress was associated with greater challenge appraisal tendencies through more proactive coping. In turn, a challenge appraisal tendency related to greater vitality, which related to lower depressive symptoms. Results add to the growing amount of literature which support the importance of beliefs in psychological wellbeing (e.g., Turner et al., 2019). In particular, results of the present study extend the findings of previous studies in the relationship between stress mindset and challenge appraisal tendencies is mediated by proactive coping. Based on the TCTSA-R (Meijen et al., 2020), this may be because individuals who hold these beliefs about stress may feel better equipped to deal with stressful situations through a perception of greater personal resources. In turn, this chain of beliefs and cognitions may lead to greater vitality and lower depressive symptoms. Results also provide the first known evidence that athletes may hold a greater stress mindset than non-athletes, offering support for the experience of moderately stressful experiences and stress inoculation as a potential method for enhancing positive beliefs about stress.

## Data availability statement

The raw data supporting the conclusions of this article will be made available by the authors, without undue reservation.

## Ethics statement

The studies involving human participants were reviewed and approved by University of Birmingham ERN19_1194. The patients/participants provided their written informed consent to participate in this study.

## Author contributions

All authors listed have made a substantial, direct, and intellectual contribution to the work and approved it for publication.

## Conflict of interest

The authors declare that the research was conducted in the absence of any commercial or financial relationships that could be construed as a potential conflict of interest.

## Publisher’s note

All claims expressed in this article are solely those of the authors and do not necessarily represent those of their affiliated organizations, or those of the publisher, the editors and the reviewers. Any product that may be evaluated in this article, or claim that may be made by its manufacturer, is not guaranteed or endorsed by the publisher.
